# Supplementary material for: Integrated Transcriptome and Proteome Analyses Reveal the Regulatory Role of miR-146a in Human Limbal Epithelium via Notch Signaling
Source: Cells. 2020 Sep 26;9(10):2175. doi: 10.3390/cells9102175 (PMC7650592; doi:10.3390/cells9102175)
Supplement: Supplementary file 1 [file cells-09-02175-s001.zip › cells-886180 Supplementary Materials.pdf]

Supplementary Figures

Integrated Transcriptome and Proteome Analyses Reveal the Regulatory Role of miR-146a in Limbal Epithelium via Notch Signaling

Adam J. Poe [1,2], Mangesh Kulkarni [1,2], Aleksandra Leszczynska [1,2], Jie Tang [3], Ruchi Shah[1,2], Yasaman Jami-Alahmadi [4], Jason Wang [1,2], Andrei A. Kramerov [1,2], James Wohlschlegel [4], Vasu Punj [5], Alexander V. Ljubimov [1,2,6] and Mehrnoosh Saghizadeh [1,2,6]*

[1]Board of Governors Regenerative Medicine Institute, Eye Program, Departments of [2]Biomedical Sciences, and [3]Genomics Core, Cedars-Sinai Medical Center, [4]Departments of Biological Chemistry, and [5]Department of Medicine, Keck School of Medicine, University of Southern California,[6]David Geffen School of Medicine at UCLA, and, Los Angeles, CA, USA.

Supported by NIH R01 grants EY025377, EY029829 (MS), EY013431 (AVL), and grants from the Board of Governors Regenerative Medicine Institute

* Corresponding author. Dr. Mehrnoosh Saghizadeh, E-mail: ghiamm@cshs.org

Eye Program, Regenerative Medicine Institute, Cedars-Sinai Medical Center, 8700 Beverly Boulevard, AHSP-A8104, Los Angeles, CA 90048, USA. Tel. 1-310-248-8696, E-mail ghiamm@cshs.org

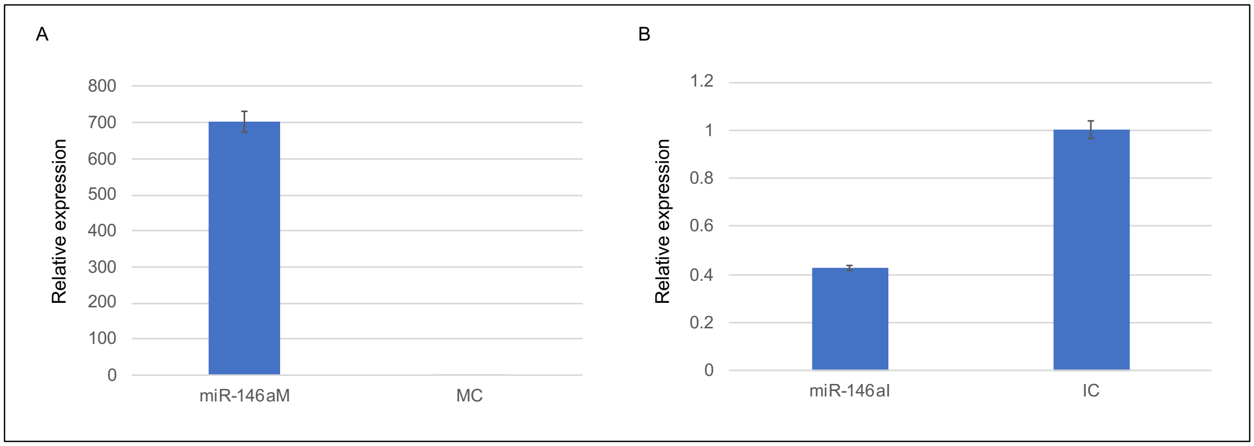

Supplementary Figure S1. Quantitative RT-PCR (QRT-PCR) validation of miR-146a expression level in transfected primary LECs. Relative expression of miR-146a in mimic- (A) and inhibitor - transfected (B) primary LECs showed its upregulation and downregulation, respectively, to their corresponding controls according to QRT-PCR. miR-146a mimic (miR-146aM), mimic control (MC), miR-146a inhibitor (miR-146aI), inhibitor control (IC).

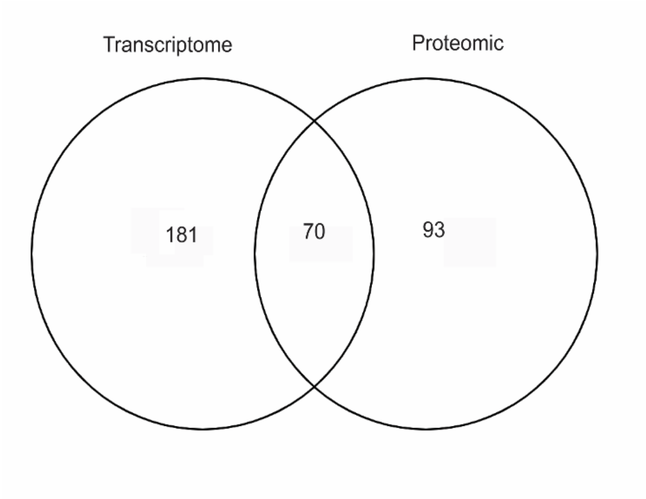

Supplementary Figure S2. Comparison of differentially expressed genes/protein in the integrated transcriptome and proteomics dataset. **The** Venn diagram of two-group comparisons of differentially expressed genes/proteins (FDR p < 0.05, Fold Change ±1.2) in the integrated transcriptome and proteomics dataset showed 70 overlapping expressed targets.

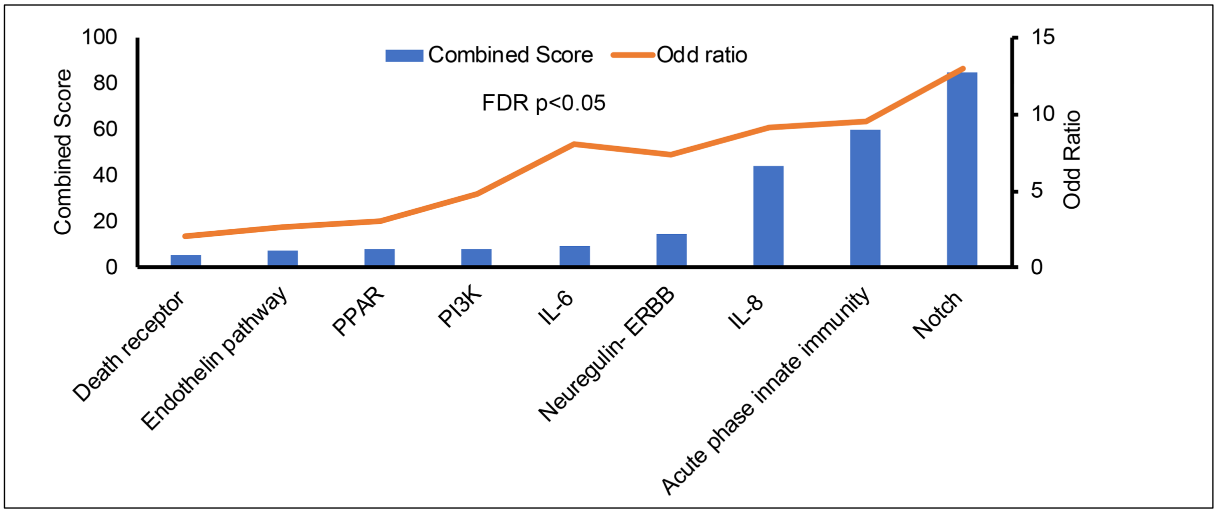

Figure S3. Identification of the overall most significantly altered pathways in RNA-seq in miR-146aM- compared to mimic-control-transfected LECs. Pathways are ranked based on the combined enrichment score and Odd ratio. Notch is one of the most significantly altered pathways (FDR *p* <0.05), with a combined enrichment score of 85 and Odd ratio of 12 among the differentially enriched pathways.

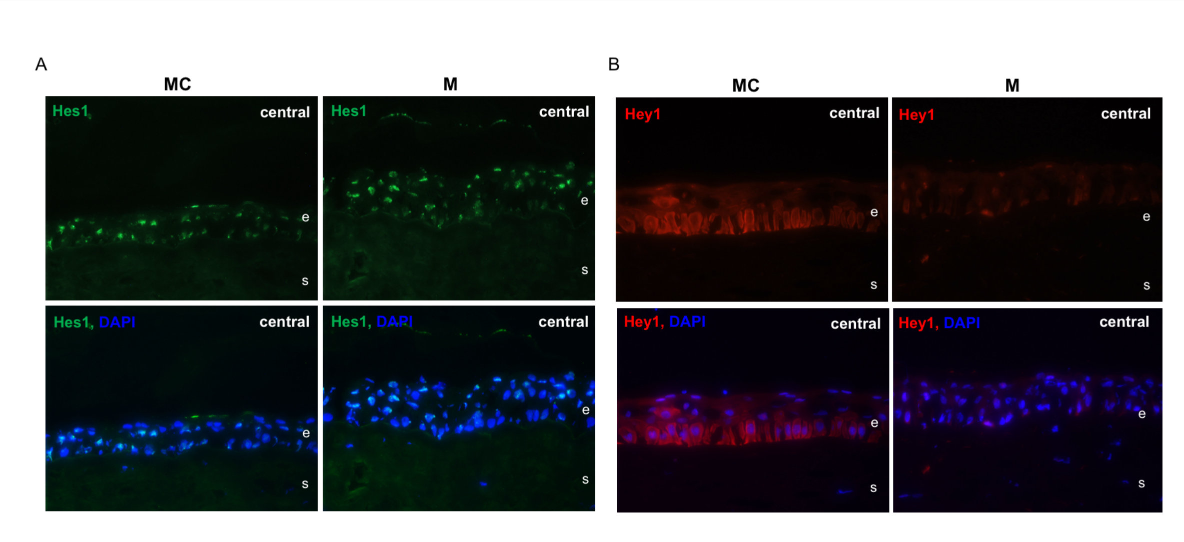

Supplementary Figure S4. Effect of miR-146a on the expression of downstream targets of Notch signaling in human organ-cultured central corneas with immunostaining. Corneas transfected with miR-146a mimic (M) showed no difference in expression of Hes1 (A) and decreased expression of Hey1 (B) in the central corneal epithelium compared to their mimic control (MC)-transfected fellow corneas.
